# Comorbid psychiatric disorders and long-term survival after liver transplantation in transplant facilities with a psychiatric consultation-liaison team: a multicenter retrospective study

**DOI:** 10.1186/s12876-023-02735-1

**Published:** 2023-04-05

**Authors:** Hiroyuki Kimura, Shinichi Kishi, Hisashi Narita, Teruaki Tanaka, Tsuyoshi Okada, Daisuke Fujisawa, Naoko Sugita, Shun’ichi Noma, Yosuke Matsumoto, Ayako Ohashi, Hiroshi Mitsuyasu, Keizo Yoshida, Hiroaki Kawasaki, Katsuji Nishimura, Yasuhiro Ogura, Norio Ozaki

**Affiliations:** 1 Department of Psychiatry, Nagoya University Graduate School of Medicine, 65 Tsurumai-Cho, Showa-Ku, Nagoya, 466-8560 Japan; 2 Department of Psychiatry, Hokkaido University Graduate School of Medicine, Kita14, Nishi5, Kita-Ku, Sapporo, 060-8648 Japan; 3 Department of Psychiatry, KKR Sapporo Medical Center, 1-6-3-40, Hiragishi, Toyohira-Ku, Sapporo, 062-0931 Japan; 4 Department of Psychiatry, Jichi Medical University, 3311-1 Yakushiji, Shimotsuke-Shi, Tochigi, 329-0498 Japan; 5 Department of Neuropsychiatry, Keio University Graduate School of Medicine, 35 Shinanomachi, Shinjuku-Ku, Tokyo, 160-8582 Japan; 6 Department of Psychiatry, Graduate School of Medicine, Kyoto University, 54 Shogoin-Kawahara-Cho, Sakyo-Ku, Kyoto, 606-8507 Japan; 7 Okayama University Hospital Gender Center, 2-5-1 Shikata-Cho, Kita-Ku, Okayama, 700-8558 Japan; 8 Department of Neuropsychiatry, Graduate School of Medical Sciences, Kyushu University, 3-1-1, Maidashi, Higashi-Ku, Fukuoka, 812-8582 Japan; 9 Department of Psychiatry, Tanushimaru Central Hospital, 892 Masuoda, Tanushimaru, Kurume, Fukuoka, 839-1213 Japan; 10 Kariya Yoshida Mental Clinic, 2F, FBterrace Bldg, 2-40 Aioi-Cho, Kariya-Shi, Aichi 448-0027 Japan; 11 Department of Psychiatry, Faculty of Medicine, Fukuoka University, 7-45-1 Nanakuma, Jonan-Ku, Fukuoka, 814-0180 Japan; 12 Department of Psychiatry, Tokyo Women’s Medical University School of Medicine, 8-1 Kawada-Cho, Shinjyuku-Ku, Tokyo, 162-8666 Japan; 13 Department of Transplantation Surgery, Nagoya University Hospital, 65 Tsurumai-Cho, Showa-Ku, Nagoya, 466-8560 Japan; 14 Institute for Glyco-Core Research (iGCORE), Nagoya University, Furo-Cho, Chikusa-Ku, Nagoya, 464-8601 Japan

**Keywords:** Liver transplant recipient, Psychiatric disorder, Survival rate, Consultation-liaison psychiatry

## Abstract

**Objective:**

Comorbid psychiatric disorders negatively affect the survival rate of patients with some physical disorders. In liver transplant recipients, various psychiatric disorders have been identified as worsening prognosis. However, little is known about how the presence of any comorbid (overall) disorders affect the survival rate of transplant recipients. In this study, we examined the effect of overall comorbid psychiatric disorders on survival rate in liver transplant recipients.

**Methods:**

A total of 1006 recipients who underwent liver transplantation between September 1997 and July 2017 across eight transplant facilities with a psychiatric consultation-liaison team were identified consecutively. Recipients were categorized into those with comorbid psychiatric disorders and those without comorbid psychiatric disorders. In the comorbid psychiatric disorder group, psychiatric disorder diagnosis and time of diagnosis were investigated retrospectively.

**Results:**

Of the 1006 recipients, 294 (29.2%) had comorbid psychiatric disorders. Comorbid psychiatric disorders in the 1006 recipients were insomnia (*N* = 107, 10.6%), delirium (*N* = 103, 10.2%), major depressive disorder (*N* = 41, 4.1%), adjustment disorder (*N* = 19, 1.9%), anxiety disorder (*N* = 17, 1.7%), intellectual disability (*N* = 11, 1.1%), autism spectrum disorder (*N* = 7, 0.7%), somatic symptom disorder (*N* = 4, 0.4%) schizophrenia (*N* = 4, 0.4%), substance use disorder (*N* = 24, 2.4%) and personality disorder (*N* = 2, 0.2%). The most common time of psychiatric disorder diagnosis was within the first 3 months after liver transplantation (51.6%). The final mortality in patients with comorbid psychiatric disorder diagnosis during the five periods (pretransplant, transplant to 3 months, months to 1 year, 1 to 3 years, and over 3 years posttransplant) was 16.2%, 18.8%, 39.1%, 28.6%, and 16.2% respectively, and there were no significant differences between the five periods (χ2 = 8.05, df = 4, *p* = 0.09). Overall comorbid psychiatric disorders were significantly associated with shorter survival time (log-rank test: *p* = 0.01, hazard ratio: 1.59 [95% confidence interval: 1.14–2.21], survival rate at the endpoint [%]: 62.0 vs. 83.3). However, after adjusting for confounding variables using Cox proportional hazards regression, there was no significant effect of overall comorbid psychiatric disorders on prognosis.

**Conclusion:**

Comorbid psychiatric disorders did not affect the survival rate of liver transplant recipients in this study.

## Background

Comorbid psychiatric disorders negatively affect the prognosis of several physical disorders, including coronary heart disease, stroke, and diabetes [1]. Many studies have examined the effect of comorbid psychiatric disorders on transplant recipients. These studies collectively demonstrate that approximately 40% of transplant recipients have psychiatric disorders pretransplantation [2], and that approximately 60% of organ recipients develop psychiatric disorders within several years posttransplantation [3]. A comprehensive meta-analysis of comorbid psychiatric disorders in organ transplant recipients [3] demonstrated that recipients who present with depression prior to and immediately after transplantation have 1.65 times the risk of postoperative mortality compared with those without comorbid psychiatric disorders. Because of such findings, comorbid psychiatric disorders are carefully taken into consideration as possible contraindications to organ transplantation. For example, the American Association for the Study of Liver Diseases practice guidelines [4] recommend that psychiatric disorders that are poorly controlled by appropriate pharmacotherapy should be considered relative contraindications to transplantation, and the clinical guidelines of the British Society of Gastroenterology [5] indicate that severe psychiatric disorders are relative contraindications to transplantation.

Liver transplant recipients often have comorbid psychiatric disorders that are insufficiently severe to meet the above criteria for contraindications. Depression is the most examined psychiatric disorder, and has been reported to be comorbid in 13%–60% of liver transplant recipients [6–9]. Furthermore, pretransplant depression reportedly reduces the quality of life and survival of transplant recipients [8]. Studies [10, 11] have also demonstrated that 10%–27% of liver transplant recipients have delirium, and that these recipients are at higher risk of prolonged hospital length of stay and mortality [12, 13]. Similarly, 5%–6% of liver transplant recipients, particularly adolescent recipients, have posttraumatic stress disorder [14, 15]. Recipients with various types of psychiatric disorders are treated by psychiatrists who provide multidisciplinary care in the field of transplant medicine. Studies [7, 16] have demonstrated that psychiatric intervention improves long-term survival in liver transplant recipients.

Clinicians often assume that psychiatric disorders may lead to low adherence and consequently poor long-term survival rate in transplant recipients. However, no studies have examined the presence of any psychiatric (overall) disorder in liver transplant recipients. The prevalence of psychiatric disorders is high, they are often comorbid, and they affect prognosis in varying degrees. Prevention of any psychiatric disorder would be important to improve prognosis. From this perspective, the categorization of any psychiatric (overall) disorder is of certain significance.

In the present study, we investigated the prevalence of comorbid psychiatric disorders in liver transplant recipients, and whether both overall and specific psychiatric comorbidities affected their long-term survival.

## Methods

### Patients and study design

The Organ Failure/Transplantation Committee of the Japanese Society of General Hospital Psychiatry, which consists of psychiatrists who specialize in transplant medicine, as well as psychologists, recipient coordinators, and nurses, selected eight institutions (Hokkaido University, Jichi University, Keio University, Tokyo Women's Medical University, Nagoya University, Kyoto University, Okayama University, and Kyushu University). These eight facilities were selected because of having a number of liver transplants and proven consultation-liaison psychiatry programs that include transplant surgeons and psychiatrists. Activities of the liaison team at each facility have been gradually standardized since 2009 due to the development of the Organ Failure/Transplantation Committee of the Japanese Society of General Hospital Psychiatry.

During the pretransplantation period, the transplant team consulted with the expert psychiatrists and psychologists belonging to the transplant psychiatry team regarding any recipient candidates with a history of psychiatric disorders or psychiatric signs and symptoms. As per the transplantation criteria in Japan, recipient candidates with liver cirrhosis owing to alcohol use disorders were disqualified from transplant listing if they had not been abstinent for the last 6 months (on February 20, 2014, this changed to 18 months), indicating that they were mentally stable before liver transplantation.

During the posttransplantation period, the transplant team consulted with the expert psychiatrists and psychologists belonging to the transplant psychiatry team if they noticed any signs or symptoms of psychiatric disorders in recipients. Following requests from the transplant team during each period, expert psychiatrists and psychologists at each institution evaluated all patients for comorbid psychiatric disorders using semi-structured interviews based on the Diagnostic and Statistical Manual of Mental Disorders (DSM)-IV [17], DSM-IV-TR [18], and DSM-5 [19].

A total of 1006 recipients, who underwent liver transplantation between September 1997 and July 2017 across the institutions, were included consecutively in this study. For this study, diagnoses identified by diagnostic criteria prior to DSM-5 were changed to those corresponding approximately to diagnoses by DSM-5. After the data collection period, data were extracted by experienced psychiatrists and psychologists who were part of the transplant psychiatry team of each institution. Electronic patient records were used to collect clinical data for all transplant recipients (age at transplant, sex, marital status, education, model for end-stage liver disease (MELD)/pediatric end-stage liver disease (PELD), etiology of liver disease, living donor liver transplantation/deceased donor liver transplantation, relationship of recipient to donor, hospitalization days from transplantation to leaving the hospital, death). Data on psychiatric comorbidity, including time of diagnosis, were also collected, and recipients were categorized into those with comorbid psychiatric disorders (comorbid psychiatric disorders group: CP group) and those without comorbid psychiatric disorders (non-comorbid psychiatric disorders group: N-CP group). All the data were aggregated at Nagoya University before undergoing statistical processing.

First, demographics, liver disease factors, and transplant factors were compared between the CP and N-CP groups. Second, based on a previous study of comorbid psychiatric disorders [20], recipients in the CP group were further categorized according to the time of psychiatric disorder diagnosis (pretransplant, transplant to 3 months, 3 months to 1 year, 1 to 3 years, and over 3 years posttransplant) to compare the mortality between these subgroups. Third, the survival rate was compared between the CP and N-CP groups. According to one definition of delirium [19], there is evidence that such disturbances are a “direct physiological consequence” of another medical condition, substance intoxication or withdrawal (i.e., caused by drug abuse or medication), toxins, or various combinations of causes. As the substantial physical effect of the transplant procedure may have led to delirium during the first week after liver transplantation [12, 21], the same analyses were also performed excluding patients with postoperative delirium from the CP group to control for the acute, direct physiological effect of liver transplantation. And the same analysis was then performed excluding all delirium patients.

Furthermore, the survival rate in each comorbid psychiatric disorder group was compared with that of the N-CP group as a comparison with previous findings [8, 13]. Survival was analyzed separately for participants aged 12 years or older (using MELD) and in those aged less than 12 years (using PELD), because the nature and distribution of psychiatric comorbidity differs between these two groups. Finally, psychiatric disorders were classified into major groups (schizophrenia, major depressive disorder, mild intellectual disability, and autism spectrum disorder), minor groups (insomnia, adjustment disorder, and anxiety disorder), and other groups (substance use disorder and personality disorder), and mortality rates at 1 and 3 years after transplant were tested for comparison.

Hospitalization day data were not available for 18 of the 1006 recipients. Additionally, MELD data were not available for 161 of the 669 recipients aged 12 years or older. These participants were therefore excluded from analyses that required these data.

### Statistical analysis

Statistical analyses were performed using IBM SPSS statistics version 25 (IBM, Armonk, NY, USA) and EZR [22]. Continuous variables were compared using Student’s t-tests and categorical variables using the chi-square test or Fisher’s exact test as appropriate. Residual analysis was performed when the cross-tabulation table was greater than 2 × 2. For comparisons of patient characteristics, the significance level was set at 0.05 and not adjusted for multiple testing because of the exploratory nature of the study.

Time to death was estimated using Kaplan–Meier curves. Comparisons of the two survival curves were performed using the log-rank test. Data for groups with/without overall psychiatric disorder or each psychiatric disorder, with a power of approximately 0.8 or higher under a type 1 error of 0.01 or 0.025 where appropriate, were subjected to Kaplan–Meier analysis using the log-rank test.

Cox proportional hazards regression analysis was used to estimate the hazard rates (HR) for mortality, adjusting for background factors. The significance level was adjusted for multiple comparisons as appropriate.

## Results

Table 1 shows the demographic characteristics of liver transplant recipients. The CP group contained more participants who were older, married, had more years of education, higher MELD score, and longer hospitalization than the N-CP group. Table 2 shows diagnosis and time of diagnosis of psychiatric disorders in the CP group. Multiple comorbidities at the same time were recorded separately. Only the first episode was recorded in patients having relapses of a single disorder. Every diagnosis was recorded in patients having more than one disorder over time. Comorbid psychiatric disorders in the 1006 recipients were insomnia (*N* = 107, 10.6%), delirium (*N* = 103, 10.2%), major depressive disorder (*N* = 41, 4.1%), adjustment disorder (*N* = 19, 1.9%), anxiety disorder (*N* = 17, 1.7%), intellectual disability (*N* = 11, 1.1%), autism spectrum disorder (*N* = 7, 0.7%), somatic symptom disorder (*N* = 4, 0.4%) schizophrenia (*N* = 4, 0.4%), substance use disorder (*N* = 24, 2.4%) and personality disorder (*N* = 2, 0.2%).

**Table 1:** Patient characteristics for the cohort and by psychiatric disorder status

Patients characteristic	Total	No Comorbid Psychiatric disorders Group	Comorbid Psychiatric disorders Group	*P* Value
age at transplant: mean (age) ± SD	33.37 ± 25.03	27.86 ± 25.39	46.70 ± 18.31	< .001**
sex ( N)				
female	551	398	153	.26
male	455	314	141	
marital status (N)				
married	467	273	194	< .001**
single	458	387	71	< .001**
unknown	81	52	29	.16
education: mean (year) ± SD	6.05 ± 6.21	4.73 ± 5.94	10.41 ± 4.99	< .001**
liver disease factors				
MELD: mean ± SD	17.85 ± 9.12	17.14 ± 9.06	18.94 ± 9.14	.04*
PELD: mean ± SD	11.67 ± 9.46	11.75 ± 9.46	10.65 ± 9.91	.628
etiology of liver disease (N)				
BA	297	267	30	< .001**
HCV-LC	136	82	54	.05*
HCC	89	60	29	.549
PBC	101	57	44	.003**
fulminant hepatitis	72	45	27	.13
PSC	34	24	10	< .99
ALD-LC	47	18	29	< .001**
AIH	2	0	2	.03*
other	351	239	112	.317
transplant factors				
DBD donor (N)	27	18	11	.3
living donor (N)	979	694	283	
relationship of recipient to donor ( N)				
parent and child	557	405	152	.134
spouse	256	177	79	.484
sibling	88	60	28	.549
other	57	37	20	.317
unknown	48	33	15	.764
hospitalization days: mean ( days) ± SD	64.63 ± 60.16	58.44 ± 50.49	79.46 ± 76.60	< .001**
life or death (N)				
alive	857	618	239	.03*
dead	149	94	55	

*SD* Standard deviation, *N* Number, *MELD* Model for end-stage liver disease, *PELD* Pediatric end stage liver disease, *BA* Biliary atresia, *HCV-LC* C-type cirrhosis, *HCC* Hepatocellular carcinoma, *PBC* Primary biliary cholangitis, *PSC* Primary sclerosing cholangitis, *ALD-LC* Alcoholic cirrhosis, *AIH* Autoimmune hepatitis, *DBD donor* Donation after brain death donor, hospitalization days, days from donation to leaving the hospital

^*^p < .05, **p < .01

**Table 2:** Time of diagnosis of psychiatric disorders

Psychiatric disorders(n)	Overall	Pre-transplant	Transplant to 3 months	3 months to 1 year	1 year to 3 years	Over 3 years
insomnia	107	30(3.0)	69(7.1)	4(0.4)	2(0.2)	2(0.2)
delirium	103	15(1.5)	78(8.0)	5(0.5)	0(0.0)	5(0.6)
depression	41	14(1.4)	16(1.6)	6(0.6)	3(0.3)	2(0.2)
substance use disorder	24	21(2.1)	0(0.0)	1(0.1)	1(0.1)	1(0.1)
adjustment disorder	19	3(0.3)	8(0.8)	1(0.1)	1(0.1)	6(0.7)
anxiety disorder	17	5(0.5)	5(0.5)	2(0.2)	4(0.4)	1(0.1)
intellectual disability	11	2(0.2)	0(0.0)	0(0.0)	0(0.0)	9(1.0)
autism spectrum disorder	7	2(0.2)	0(0.0)	0(0.0)	0(0.0)	5(0.6)
somatoform disorder	4	1(0.1)	1(0.1)	1(0.1)	0(0.0)	1(0.1)
schizophrenia	4	1(0.1)	0(0.0)	0(0.0)	1(0.1)	2(0.2)
personality disorders	2	2(0.2)	0(0.0)	0(0.0)	0(0.0)	0(0.0)
Other	28	0	14(1.4)	3(0.3)	2(0.2)	9(1.0)
Survivor		0	978/1006	929/1006	913/1006	898/1006

Number of survivors after transplantation was defined as the number of survivors at the middle of the period, and for Over 3 years the number of survivors at 3 years

Figures in parentheses represent the percentage of new cases to survivors at that time

The most common time of psychiatric disorder diagnosis was within the first 3 months after liver transplantation (51.6%). The final mortality in patients with comorbid psychiatric disorder diagnosis during the five periods (pretransplant: A, transplant to 3 months: B, months to 1 year: C, 1 to 3 years: D, and over 3 years posttransplant: E) was 16.2%, 18.8%, 39.1%, 28.6%, and 16.2% respectively, and there were no significant differences between the five periods (χ2 = 8.05, df = 4, *p* = 0.09). Additional cox proportional hazards regression revealed that the period of first onset psychiatric disorder did not affect survival outcome (A: *p* = 0.19, HR: 2.37[95% CI: 0.66–8.56], B: *p* = 0.06, 3.22[0.94–11.00], C: *p* = 0.11, 3.43[0.77–15.37], D: *p* = 0.72, 1.52[0.16–14.71], E: reference).

Among the groups of major (schizophrenia, major depressive disorder, mild intellectual disability, and autism spectrum disorder), minor (insomnia, adjustment disorder, and anxiety disorder), and other psychiatric disorders (substance use disorder and personality disorder), mortality rates at 1 and 3 years after transplant were not significantly different, *P* = 0.8.

### Survival outcome

Over the length of the study follow-up, 149 (14.8%) recipients died and 857 (85.2%) were alive at the end of the study. Comorbid psychiatric disorders were significantly associated with shorter survival time (log-rank test: *p* = 0.01, HR: 1.59, 95% confidence interval [CI]: 1.14–2.21). This result seems to be in agreement with the results of previous studies and to support transplant guidelines for patients with comorbid psychiatric disorders.

The same analyses performed excluding patients with delirium during the first week after transplantation also showed significant association between comorbid psychiatric disorders and shorter survival time. However, the same analysis excluding all patients with delirium did not show a significant association between comorbid psychiatric disorders and shorter survival. Delirium and major depressive disorder were significantly associated with shorter survival time, but insomnia was not (Table 3).

**Table 3:** The survival rate for each comorbid psychiatric disorder

	comorbid psychiatric disorders (n)		*p*	HR	95%CI	Survival rate at the end point(%)***		Power
	Included	Not included				Included	Not included	
comorbid psychiatric disorders	294	712	0.005**	1.585	1.135–2.214	62.0	83.3	1.00
comorbid psychiatric disorders excluding the delirium within 1 week after transplant	242	712	0.010*	1.550	1.085–2.213	61.6	83.3	0.99
comorbid psychiatric disorders excluding the delirium	191	712	0.631	1.112	0.720–1.719	66.3	83.3	0.91
insomnia	107	712	0.370	1.267	0.733–2.192	58.9	83.3	0.93
delirium(Overall)	103	712	< 0.001**	2.459	1.620–3.735	54.4	83.3	0.97
major depressive disorder	41	712	< 0.001**	2.635	1.503–4.621	43.8	83.3	0.91

^*^p < .01.** *p* < .001. ***Obtained by Kaplan–Meier survival curves. The significance level was set at *p* < .01, which was calculated by dividing .05 by the number of comparisons. Groups with a power of approximately .8 or higher under a type 1 error of .01 were subjected to the analyses

*HR* Hazard ratio, *CL* Confidence interval

There were differences in background characteristics between the CP group and the N-CP group (Table 1). Cox proportional hazards regression was performed with data for all transplant recipients to adjust for these differences (Table 4). Age, hospitalization days, and MELD score were the main factors that showed significant differences between the CP and N-CP groups. In addition, since this study analyzed psychiatric disorders of various onset times together, a factor of time of diagnosis (time from onset to transplantation) was added. In the case of multiple psychiatric disorders, the date of diagnosis of the first psychiatric disorder was selected. In the case of preoperative psychiatric disorder, since the date of onset of psychiatric disorder was unknown, the date of onset of psychiatric disorder was tentatively set to one month before transplantation, when the preoperative evaluation was performed. As MELD score only applied to recipients aged 12 years or older, age and hospitalization days were first entered as covariates for all transplant recipients. The results showed that age and hospitalization days had a significant effect on survival (age: *p* < 0.001, HR: 1.03 [95% CI: 1.02–1.04]; hospitalization days: *p* < 0.001, HR: 1.004 [95% CI: 1.003–1.006]), whereas comorbid psychiatric disorders had no significant effect (comorbid psychiatric disorders: *p* = 0.75, HR: 0.94 [95% CI: 0.66–1.35]). The same analyses performed for all transplant recipients excluding those with delirium during the first week after transplantation also showed no significant association between comorbid psychiatric disorders and shorter survival time. Furthermore, the same analysis was performed for each comorbid psychiatric disorder. The results showed that delirium(Overall), insomnia, and major depressive disorder did not significantly affect survival.

**Table 4:** Cox proportional hazards regression adjusting age and hospitalization days

Covariate	HR	95%cl	*p*
comorbid psychiatric disorders			
comorbid psychiatric disorders	0.943	0.657–1.353	0.749
age	1.032	1.023–1.041	< .001**
hospitalization days	1.004	1.003–1.006	< .001**
time of diagnosis	0.989	0.965–1.012	0.337
comorbid psychiatric disorders excluding delirium within 1 week			
comorbid psychiatric disorders excluding 1 week	0.791	0.644–1.399	0.791
age	1.032	1.023–1.041	< 0.001**
hospitalization days	1.004	1.002–1.006	< 0.001**
time of diagnosis	0.989	0.966–1.012	0.351
comorbid psychiatric disorders excluding delirium			
comorbid psychiatric disorders excluding delirium	0.675	0.416–1.094	0.111
age	1.032	1.023–1.042	< 0.001**
hospitalization days	1.004	1.003–1.006	< 0.001**
time of diagnosis	0.990	0.964–1.016	0.439
delirium(Overall)			
delirium	1.310	0.850–2.020	0.221
age	1.033	1.024–1.043	< 0.001**
hospitalization days	1.004	1.002–1.007	< 0.001**
time of diagnosis	1.000	0.974–1.026	0.994
insomnia(Overall)			
insomnia	0.635	0.356–1.135	0.125
age	1.034	1.024–1.044	< 0.001**
hospitalization days	1.004	1.022–1.007	< 0.001**
time of diagnosis	0.996	0.967–1.026	0.783
major depressive disorder(Overall)			
major depressive disorder	1.249	0.658–2.369	0.497
age	1.035	1.024–1.045	< 0.001**
hospitalization days	1.003	1.001–1.005	0.001**
time of diagnosis	0.992	0.962–1.023	0.597

The significance level was set at *p* < .01, which was calculated by dividing .05 by the number of comparisons

*HR* hazard ratio, *Cl* Confidence interval

^*^p < .01. **p < .001

Similarly, survival was analyzed separately in participants aged 12 years or older (using MELD) and in those aged less than 12 years (using PELD).

In participants aged 12 years or older, there was sufficient power for an analysis of comorbid psychiatric disorders and comorbid psychiatric disorders excluding delirium during the first week after transplantation. In participants aged less than 12 years, there was insufficient power for an analysis of each diagnostic group; therefore, additional analyses after separating by age were not performed.

Kaplan–Meier analysis showed that comorbid psychiatric disorders (including/excluding delirium during the first week after transplantation) in participants aged 12 years or older were not significantly associated with survival time (Table 5). Cox proportional hazards regression analysis showed that age and hospitalization days had a significant effect on survival, whereas comorbid psychiatric disorders (including/excluding delirium during the first week after transplantation) and MELD had no significant effect (Table 6). Delirium can affect the prognosis of liver transplantation recipients, resulting in longer hospitalization. Including both delirium and hospitalization day in covariates might be problematic in terms of multicollinearity. Therefore, Cox proportional hazards regression was performed excluding the covariate of hospitalization days. As a result, the survival rate was not affected by the comorbidity of psychiatric disorders(data not shown). Without adjusting for background factors, delirium, and major depressive disorder appeared to be significantly associated with shorter survival in this study. However, further Cox proportional hazards regression analysis with sufficient power showed no association, indicating no impact of delirium and major depressive disorder on survival.

**Table 5:** The survival rate for each comorbid psychiatric disorder (aged 12 years and older)

	comorbid psychiatric disorders (n)		*p*	HR	95%CI	Survival rate at the end point(%)^a^		Power
	Included	Not included				Included	Not included	
comorbid psychiatric disorders	270	399	0.774	1.052	0.745–1.486	56.1	71.2	0.83
comorbid psychiatric disorders excluding the delirium within 1 week after transplant	218	399	0.797	1.050	0.727–1.516	54.5	71.2	0.83

*HR* Hazard ratio, *Cl* Confidence interval

^a^Obtained by Kaplan–Meier survival curves. The significance level was set at *p* < ,025, which was calculated by dividing .05 by the number of comparisons. Groups with a power of approximately 0.8 or higher under a type 1 error of .025 were subjected to the analyses

**Table 6:** Cox proportional hazards regression adjusting age, hospitalization days and MELD

Covariate	HR	95%cl	*p*
comorbid psychiatric disorders			
comorbid psychiatric disorders	1.148	0.761–1.731	0.511
age	1.019	1.003–1.034	0.019*
hospitalization days	1.005	1.003–1.007	< 0.001**
MELD	1.009	0.988–1.030	0.406
time of diagnosis	0.991	0.963–1.020	0.540
comorbid psychiatric disorders excluding delirium within 1 week			
comorbid psychiatric disorders excluding the delirium within 1 week	1.181	0.767–1.819	0.450
age	1.018	1.002–1.035	0.026
hospitalization days	1.005	1.002–1.007	< 0.001**
MELD	1.008	0.986–1.031	0.491
time of diagnosis	0.991	0.963–1.020	0.534
comorbid psychiatric disorders excluding delirium			
comorbid psychiatric disorders excluding the delirium	0.703	0.420–1.178	0.181
age	1.019	1.003–1.035	0.016*
hospitalization days	1.005	1.003–1.007	< 0.001**
MELD	1.010	0.989–1.030	0.352
time of diagnosis	0.997	0.971–1.024	0.830

The significance level was set at *p* < .01, which was calculated by dividing .05 by the number of comparisons. *MELD* Model for end-stage liver disease, *HR* Hazard ratio, *Cl* Confidence interval. Cox proportional hazards regression excluding the covariate of hospitalization days showed that psychiatric comorbidity had no effect on survival (data not shown)

^*^p < .025. **p < .001

In conclusion, comorbid psychiatric disorders had no significant effect on survival of liver transplant recipients.

## Discussion

At first glance, the results of this study may provide a picture of the relationship between comorbid psychiatric disorders and transplant outcomes as envisioned by transplant physicians in light of previous studies and transplant guidelines for patients with comorbid psychiatric disorders. However, after adjusting for background factors, this study showed that the presence of overall as well as specific comorbid psychiatric disorders in liver transplant recipients did not affect their long-term survival. Similar results were obtained in the analyses for selected participants aged 12 years or older.

Previous studies have examined the effect of depression and delirium on the survival rate of liver transplant recipients. A prospective cohort study demonstrated that pretransplant depression was associated with poor long-term survival [7, 8], and there is also evidence that delirium is associated with mortality [13].

In considering why psychiatric disorders affect survival, we can assume that recipients with comorbid psychiatric disorder have low adherence and subsequent low long-term survival rate. Previous studies [22, 23] have demonstrated low adherence to medical treatments in recipients with psychiatric disorders. It is likely that, during a long-term follow-up, the involvement of psychiatrists in addressing comorbid psychiatric disorders becomes less frequent, leading to poor adherence and poor long-term survival. However, the comorbidity of overall psychiatric disorder, and that of each of the psychiatric disorders, had no effect on the long-term survival of transplant recipients in the present study. In previous studies, the survival rate of recipients with depression in the first posttransplant year improved when they were treated appropriately [9, 16]. It is possible that adequate long-term treatment of comorbid psychiatric disorders (perhaps by Japan’s universal health insurance program) suppressed their negative effect on the long-term survival of liver transplant recipients in this study.

In terms of methodology, almost all previous studies have compared survival rates in groups with or without one specific comorbid psychiatric disorder. In such studies, other psychiatric disorders are more likely to be present in the non-CP group. In the present study, comprehensive psychiatric disorders were diagnosed by expert psychiatrists and psychologists using standard diagnostic criteria. One previously reported large-scale study with more participants than this study lacked sufficient diagnostic accuracy [8].

Although some previous studies on depression have used the Cox proportional hazards survival approach with log-rank testing of Kaplan–Meier curves, the sample sizes were small [6, 7, 16, 24] and power analysis was not performed [6, 16, 24]. In addition, one study [9] did not include any physical indices; only age and sex were adjusted for. Moreover, for the previous studies on delirium, the sample size was small and power analysis was not performed [10, 12, 13, 21]. In the present study, appropriate power analysis was performed and background factors were investigated in detail. The use of this methodology means that the present findings may be more reliable than those of previous studies.

The most common time of diagnosis of psychiatric disorders was within the first 3 months of liver transplantation; 51.6% of psychiatric comorbidities were diagnosed in this period. This rate is higher than that reported in a previous study (20.7%) [20] in which the most common time of psychiatric disorder diagnosis was also within the first 3 months of liver transplantation. Postoperative delirium and sleep disorders likely contributed to the diagnosis of psychiatric disorders during this time period.

It is possible that the present study identified more psychiatric disorders because of the consultation-liaison psychiatry programs of the hospitals in this study; collaboration with psychiatric teams could have improved ability of transplant teams to notice psychiatric disorders. It is important to carefully monitor the development of psychiatric disorders within the first 3 months of transplantation.

In this study, we identified various comorbid psychiatric disorders in liver transplant recipients. The prevalence of major depressive disorder (4.1%) was lower than that reported in previous studies [6, 7, 24]. Anxiety disorder was previously reported in 19% of liver transplant recipients prior to surgery and 33% of transplant recipients during pretransplant and posttransplant periods [25]. In contrast, we found that only 1.7% of recipients had anxiety. The prevalence of insomnia in this study was 10.6%, whereas a previous study [26] demonstrated a higher prevalence of insomnia (40.3%). These disorders may have been overdiagnosed in some of the above-mentioned previous studies because they used questionnaires instead of standardized psychiatric diagnostic criteria. As delirium can be objectively diagnosed from recipients’ behavior, the prevalence of delirium in this study (10.2%) is consistent with that reported in previous studies (10%–27%) [10, 11].

The present study has some limitations. First, psychiatric evaluation by expert psychiatrists and psychologists was not schedule-based; it was performed in response to requests from the transplant team when they became aware of signs or symptoms of psychiatric disorders. Minor such signs may not have been detected by transplant teams consisting of non-psychiatrists and non-psychologists, though they detected more psychiatric disorders within the first 3 months of liver transplantation than in a previous study. There is a possibility that subjects with comorbid psychiatric disorders might have been included in the N-CP group.

Second, we did not investigate how many recipients with comorbid psychiatric disorders received psychiatric treatment, what type of psychiatric treatment they received, and their adherence to psychotropic medication. The major death causes underlying psychiatric disorders, such as suicide, non-medical adherence to immunosuppressants, poor personal care, accident-prone behavior were also not investigated. This lack of clinical data may have confounded the results.

Third, participants with transient psychiatric disorders and those with chronic or recurrent psychiatric disorders were not differentiated; data for both were included in the same analysis. In addition, data for participants with posttransplant early onset and those with late onset psychiatric disorders were included in the same analysis. It is likely that there was substantial variation among participants in the duration of clinically evident psychiatric disorders. Though this study revealed that the period of first onset psychiatric disorder did not affect survival outcome, it is difficult to get rid of the above issues completely. Most previous studies seem to have the same limitation.

Fourth, medical technology for liver transplantation has been improving daily over the 20 years between 1997 and 2017. The effects of such progress on the present data may have affected the findings.

Fifth, possible selection bias would be considered that transplant candidates with comorbid severe psychiatric disorders were more likely to be excluded and those with comorbid mild psychiatric disorders were selected.

## Conclusion

Comorbid psychiatric disorders did not affect survival of liver transplant recipients in the limited facilities having a number of liver transplants and proven liver transplant liaison by psychiatric teams in Japan. Further studies with a larger number of participants and well-designed protocols are needed to confirm the findings obtained in this study.

## Data Availability

The datasets used and analyzed during the current study are available only upon request to the Corresponding Author, as the data contain potentially identifying or sensitive psychosocial information(History of psychiatric disorders, comorbid psychiatric disorders and psychiatric state and so on). All the data supporting our findings are contained within the manuscript.

## Abbreviations

CI: Confidence interval
CP: Comorbid psychiatric disorders
DSM: Diagnostic and Statistical Manual of Mental Disorders
HR: Hazard rate
MELD: Model for end-stage liver disease
N-CP: Non-comorbid psychiatric disorders
PELD: Pediatric end-stage liver disease
SD: Standard deviation
